# Effects of prey of different nutrient quality on elemental nutrient budgets in *Noctiluca scintillans*

**DOI:** 10.1038/s41598-017-05991-w

**Published:** 2017-08-08

**Authors:** Shuwen Zhang, Hongbin Liu, Patricia M. Glibert, Cui Guo, Ying Ke

**Affiliations:** 1 Division of Life Science, The Hong Kong University of Science and Technology, Kowloon, Hong Kong SAR; 2 University of Maryland Center for Environmental Science, Horn Point Laboratory, P.O. Box 775, Cambridge, MD 21613 USA

## Abstract

*Noctiluca scintillans* (*Noctiluca*) is a cosmopolitan red tide forming heterotrophic dinoflagellate. In this study, we investigated its ingestion, elemental growth yield and excretion when supplied with different quality food (nutrient-balanced, N-limited and P-limited). Total cellular elemental ratios of *Noctiluca* were nearly homeostatic, but the ratio of its intracellular NH_4_^+^ and PO_4_^3−^ was weakly regulated. *Noctiluca* thus seems able to differentially allocate N and P to organic and inorganic pools to maintain overall homeostasis, and it regulated its internal N more strongly and efficiently than P. The latter was substantiated by its comparatively stable C:N ratio and compensatory feeding on N-limited prey. Using both starvation experiments and mass balance models, it was found that excretion of C, N, and P by *Noctiluca* is highly affected by prey nutritional quality. However, based on modeling results, nutrients seem efficiently retained in actively feeding *Noctiluca* for reproduction rather than directly released as was shown experimentally in starved cells. Moreover, actively feeding *Noctiluca* tend to retain P and preferentially release N, highlighting its susceptible to P-limitation. Recycling of N and P by *Noctiluca* may supply substantial nutrients for phytoplankton growth, especially following bloom senescence.

## Introduction

The concept of stoichiometric homeostatic regulation relates to how the elemental or biochemical composition of organisms is maintained or is altered in response to changes in the quality of resource supply (i.e., food quality)^1, 2^. In aquatic systems, organisms experience dynamic fluctuations in the availability of nutrient resources, and they vary considerably in their ability to maintain homeostasis as a function of environmental conditions^2, 3^. Primary producers are usually more flexible in regulating their elemental composition (e.g. C:P, C:N and N:P ratios) than most heterotrophs, which are largely constrained within a narrow range^2, 4–6^. Primary producers can often store nutrients supplied in excess, and this physiological plasticity in elemental composition of primary producers affects their quality as a food resource for heterotrophic herbivores^6–8^. It is becoming ever clearer that the availability and composition of resource elements relative to the needs of consumer puts constraints on ecological processes, such as food-web dynamics and nutrient recycling^2, 5, 9–11^. Regulation of elemental composition and consumer-driven nutrient recycling as a result of consuming nutrient-imbalanced algal food has been well documented for crustacean mesozooplankton, notably for cladocerans and copepods^2, 12–14^. However, comparatively little is known about effects of food nutrient content on heterotrophic dinoflagellates^6, 15, 16^, which also contribute to pelagic food web processes^17, 18^. Some recent studies have begun to address effects of variable prey quality on the elemental composition of heterotrophic and mixotrophic flagellates^15, 16, 18^, but compared to macrozooplankton, the effects on feeding and nutrient recycling are far less understood for these microzooplankton.

*Noctiluca scintillans* (hereafter *Noctiluca*) is a cosmopolitan red-tide forming heterotrophic dinoflagellate^19^. There are two types of *Noctiluca*, red and green^19, 20^. ‘Red’ *Noctiluca* are purely heterotrophic, and carotenoids are responsible for their orange-red color^19–21^. ‘Green’ *Noctiluca*, by contrast, contain the symbiotic prasinophyte *Protoeuglena noctilucae* which contributes to their green color, but these *Noctiluca* also conduct phagotrophy when the phytoplankton food supply is high^19, 22^. The *Noctiluca* studied in the present study was the red one. This voracious grazer feeds upon various food items, but phytoplankton are considered to be its main food items in the field^20^. Beyond its significance as a predator in determining carbon flow in marine food webs, *Noctiluca* is also an important agent of nutrient regeneration^19^. It can accumulate and regenerate large amount of dissolved inorganic nutrients (i.e. NH_4_^+^ and PO_4_^3−^)^23–26^ and more complex organic substances^26, 27^. For example, Ara *et al*.^23^ showed that when *Noctiluca* was abundant during April to July in Sagami Bay, Japan, its intracellular dissolved nutrient contents accounted for an average 49.2–63.7% for NH_4_^+^ and 39.2–63.7% for PO_4_^3−^of the total nutrient standing stocks respectively in the euphotic zone. Moreover, based on their measurement, daily NH_4_^+^ and PO_4_^3−^ supply by *Noctiluca* excretion was estimated to account for an average 50.6–85.4% and 80.5–135.8% of the daily N and P requirement for primary production in April–July in Sagami Bay, Japan^23^.

It has been shown that both the internal dissolved pools of NH_4_^+^ and PO_4_^3−^, and excretion rates of NH_4_^+^ and PO_4_^3−^ of *Noctiluca* depend on its nutritional status and growth rate^23, 28^. Previous studies have verified that consumption of nutrient-limited prey for 3 days, especially P-limited prey, significantly reduced the growth of *Noctiluca*, even though it was able to feed at a compensatory rate on those nutrient-imbalanced foods (except for P-limited *Thalassiosira weissflogii*)^29^. Disparity in the elemental composition between *Noctiluca* and its algal prey should also have important effects on elemental excretion, which, in turn has subsequent ecological consequences for marine ecosystems that are impacted by blooms of this dinoflagellate. Therefore, in the present study, we conducted a laboratory experiment of *Noctiluca* with prey with different elemental composition, supplemented by model predictions, to investigate homeostasis regulation in *Noctiluca* and the magnitude of its nutrient regeneration changes when faced with different quality food (in terms of elemental composition in stoichiometric ratios compared to the ‘Redfield’ proportions 106C:16N:1P).

## Results

### Elemental composition of *T. weissflogii*

Manipulation of media nutrients yielded distinctively different elemental composition in *T. weissflogii*. Cells grown in N or P-limited medium contained significantly lower amounts of cellular N and/or P than their nutrient-balanced counterparts (Table 1). Accordingly, the highest molar C:N ratio was found in the cells grown in N-limited medium, while the highest molar C:P and N:P ratios were detected in the cells cultured in the P-limited medium, reflecting their distinctly different nutritional quality as a food source for *Noctiluca* (ANOVA, followed by Fisher LSD post hoc tests, p < 0.05; Table 1).

**Table 1:** Carbon (C), nitrogen (N) and phosphorus (P) contents and molar stoichiometric ratios (N:P, C:N and C:P ratios) of *Thalassiosira weissflogii* grown in N-limited (-N), nutrient-balanced (f/2) and P-limited (-P) medium.

	-N	f/2	-P
C (pmol cell^−1^)	9.48 ± 0.41^b,c^	7.58 ± 0.14^a,c^	10.63 ± 0.57^a,b^
N (pmol cell^−1^)	0.49 ± 0.02^b,c^	1.23 ± 0.01^a,c^	1.03 ± 0.06^a,b^
P (fmol cell^−1^)	67.62 ± 3.45^b,c^	57.92 ± 0.59^a,c^	8.84 ± 0.48^a,b^
N:P	7.30 ± 0.47^b,c^	21.21 ± 0.32^a,c^	117.04 ± 9.54^a,b^
C:N	19.19 ± 0.12^b,c^	6.17 ± 0.09^a,c^	10.28 ± 0.08^a,b^
C:P	140.13 ± 9.40^c^	130.94 ± 2.74^c^	1203.30 ± 91.34^a,b^

Data are averages with standard deviations. Superscript letters indicate significant differences between nutrient treatments (ANOVA, Fisher LSD post hoc, p < 0.05).

### Ingestion and growth yield of *Noctiluca* on different quality food

**Figure 1:**
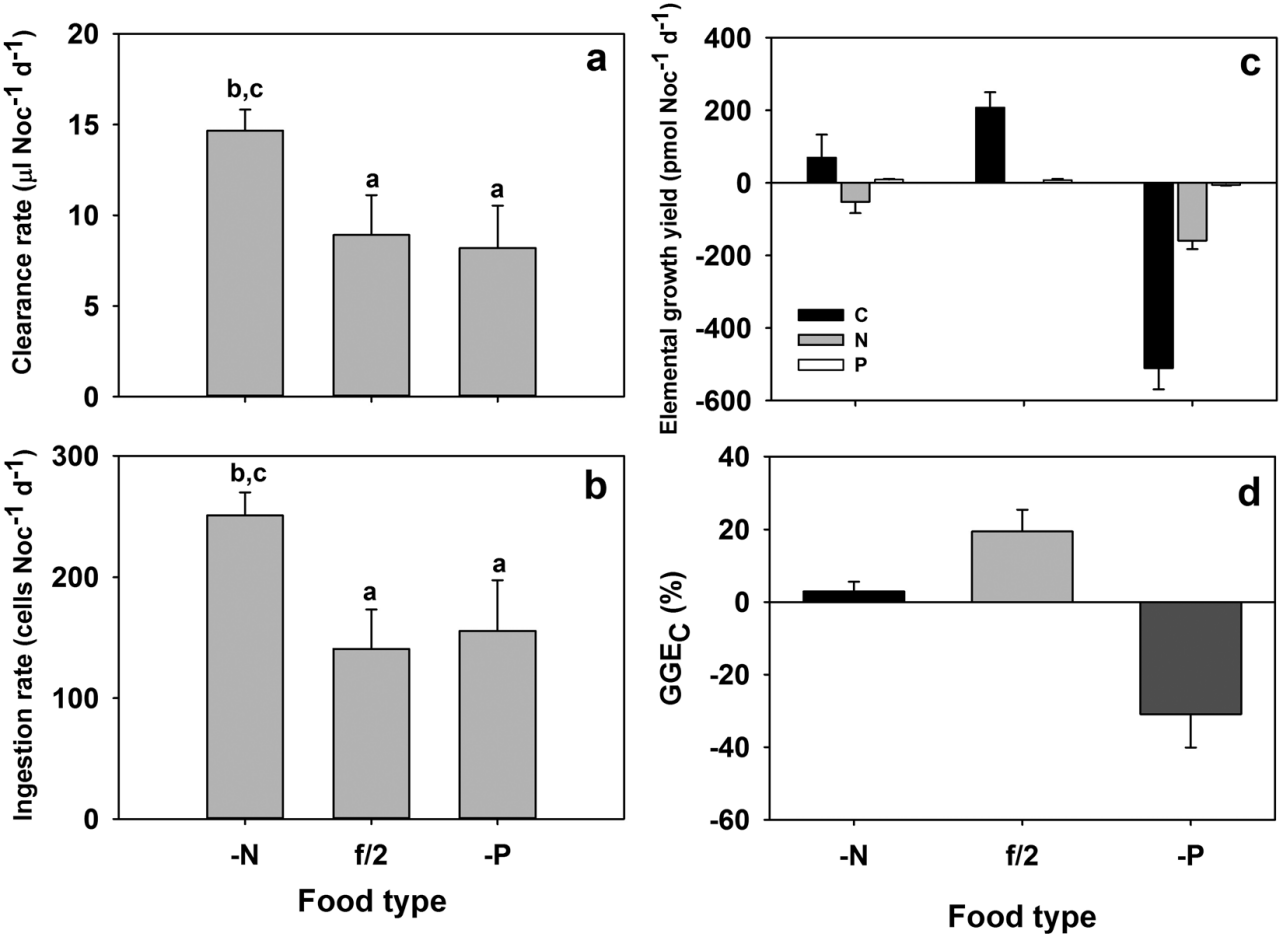
Clearance rate (**a**), ingestion rate (**b**), elemental growth yield (**c**) and gross growth efficiency (**d**) of *Noctiluca scintillans* on different quality *Thalassiosira weissflogii* [N-limited (-N), nutrient-balanced (f/2), P-limited (-P)]. Error bars = + 1 SD, n = 3. Superscript letters indicate significant differences between nutrient treatments (ANOVA, Fisher LSD post hoc, p < 0.05).

In incubations of 1 day of *Nocticula* and prey of varying nutrient content, *Noctiluca* consumed nutrient-balanced and P-limited prey with similar, but significantly lower, rates than those of N-limited prey (as clearance or ingestion rates, Fig. 1a,b; ANOVA, Fisher LSD post hoc, p < 0.05). However, regardless of amount ingested, the abundance of *Noctiluca* did not change significantly after the 1-day incubation period (Table S1; Student’s t-test, n = 6, p < 0.05). Neither did the cell diameters of *Noctiluca* change after feeding, and all had an averaged diameter around 232–240 μm in the different food treatments (Table 2). The *Noctiluca* cells collected immediately after feeding contained an average of 11, 18 and 14 *T. weissflogii* cells per individual *Noctiluca* in the N-limited, nutrient-balanced and P-limited food treatments, respectively (Table 2).

**Table 2:** Cell diameters of *Noctiluca scintillans* (µm, mean ± SD) and the number of prey held in *Noctiluca* cells (averages with the range in parenthesis) after 1d incubation with N-limited (-N), nutrient-balanced (f/2) and P-limited (-P) *Thalassiosira weissflogii*.

Treatment	Cell size (μm)	Number of prey contained in *Noctiluca*
Initial starved cell	240 ± 31	
-N	232 ± 29	11 (0~54)
f/2	235 ± 33	18 (0~78)
-P	236 ± 29	14 (0~58)

Making the assumption that over this 1 day incubation interval, prey items held in *Noctiluca* remained intact and undigested, their contribution to the elemental balance of C, N and P was small, <5% of the element contents attributed to *Noctiluca* itself. Nevertheless, the growth yield of *Noctiluca* in terms C, N, and P varied among food treatments (Fig. 1c). Those *Noctiluca* cells grown on nutrient-balanced *T. weissflogii* yielded significantly higher amounts of C, but slightly lower amounts of P (not significant) compared to those cells grown on N-limited food (Table 3, Fig. 1c; ANOVA, Fisher LSD post hoc, p < 0.05), accounting for 19.43 ± 6.03% of the C content and 95 ± 47.7% of the P content from ingestion, respectively. In contrast, consumption of P-limited prey resulted in a significant loss of elemental contents in *Noctiluca* cells; about 511 pmol C, 159 pmol N, and 5.39 pmol P were lost per *Noctiluca* in 1 d (Table 3, Fig. 1c; Student’s t-test, n = 6, p < 0.05). The gross growth efficiency based on carbon ﻿﻿(﻿GGE_C_) of *Noctiluca* was much lower on nutrient-limited prey, and was even negative on P-limited prey (2.92 ± 2.69% and −30.91 ± 9.2% on N-limited and P-limited prey respectively, Fig. 1d).

**Table 3:** The amounts and ratios of total cellular elements and intracellular NH_4_^+^ and PO_4_^3−^ of *Noctiluca scintillans* cells at the initial starved condition, after 1 day incubation with N-limited (-N), nutrient-balanced (f/2) and P-limited (-P) *Thalassiosira weissflogii*, and after subsequent 6 h starvation of those different grown cells.

Parameters	Initial	After 1 d incubation			Subsequent 6 h starvation		
		-N	f/2	-P	-N	f/2	-P
**Total cellular pools**							
C (nmol cell^−1^)	2.56 ± 0.042^c,d,B,C,D^	**2.63** ± **0.048** ^**c,d**^	**2.76** ± **0.007** ^**a,b,d**^	2.05 ± 0.04^a,b,c^	**2.08** ± **0.014** ^**a**^	**2.21** ± **0.14** ^**a**^	2.15 ± 0.069^a^
N (nmol cell^−1^)	0.46 ± 0.013^b,d,B,C,D^	**0.41** ± **0.028** ^**a,d**^	**0.43** ± **0.02** ^**d**^	**0.30** ± **0.019** ^**a,b,c**^	**0.32** ± **0.009** ^**a**^	**0.32** ± **0.023** ^**a**^	**0.34** ± **0.009** ^**a**^
P (pmol cell^−1^)	19.02 ± 1.75^b,c,d,C,D^	**27.98** ± **1.22** ^**a,d**^	**26.77** ± **2.97** ^**a,d**^	13.63 ± 1.27^a,b,c^	**22.74** ± **2.42** ^**a,D**^	**21.31** ± **0.70** ^**D**^	12.98 ± 1.16^a,B,C^
N:P	24.24 ± 2.34^b,c,B,C^	14.54 ± 1.19^a,d^	16.19 ± 1.93^a,d^	**22.35** ± **2.49** ^**b,c**^	13.86 ± 1.53^a,D^	15.06 ± 1.19^a,D^	**25.87** ± **2.40** ^**B,C**^
C:N	5.55 ± 0.19^b,c,d,B,C,D^	6.52 ± 0.47^a^	6.46 ± 0.29^a^	6.91 ± 0.44^a^	6.59 ± 0.24^a^	6.68 ± 0.37^a^	6.40 ± 0.14^a^
C:P	134.41 ± 12.59^b,c,B,C^	94.85 ± 4.46^a,d^	104.51 ± 11.51^a,d^	154.51 ± 14.74^b,c^	91.27 ± 9.73^a,D^	103.68 ± 7.44^a,D^	165.59 ± 15.65^a,B,C^
**Dissolved internal pools**							
NH_4_^+^ (pmol cell^−1^)	184.81 ± 3.71^b,c,d,B,C,D^	**130.70** ± **9.78** ^**a,c,d**^	**214.41** ± **4.68** ^**a,b,d**^	**162.35** ± **7.63** ^**a,b,c**^	**147.91** ± **9.00** ^**a,C**^	**201.23** ± **6.96** ^**a,B,D**^	**146.95** ± **1.78** ^**a,C**^
PO_4_^3−^ (pmol cell^−1^)	13.03 ± 0.24^b,c,d,B,D^	**16.75** ± **0.45** ^**a,c,d**^	**17.16** ± **0.64** ^**a,b,d**^	5.48 ± 0.37^a,b,c^	**15.24** ± **0.54** ^**a,D**^	**14.56** ± **2.37** ^**D**^	6.06 ± 1.23^a,B,C^
% NH_4_^+^ in total N pool	40.08 ± 1.42^b,c,d,B,C^	**31.95** ± **3.26** ^**a,c,d**^	**49.60** ± **2.51** ^**a,b**^	**53.81** ± **4.17** ^**a,b**^	**46.93** ± **2.60** ^**a,C**^	**62.69** ± **4.99** ^**a,B,D**^	**43.75** ± **1.25** ^**C**^
% PO_4_^3−^ in total P pool	68.48 ± 6.44^d^	59.86 ± 3.08^d^	64.10 ± 7.50^d^	40.21 ± 4.63^a,b,c^	67.02 ± 7.52	68.31 ± 11.35	46.69 ± 10.35
Molar NH_4_^+^: PO_4_^3−^	14.19 ± 0.28^b,c,d,D^	7.80 ± 0.48^a,c,d^	12.57 ± 0.43^a,b,d^	**29.66** ± **1.00** ^**a,b,c**^	9.71 ± 0.46^D^	13.82 ± 3.06^D^	**24.24** ± **5.63** ^**a,B,C**^

Superscript letters indicate significant differences between food treatments (ANOVA, Fisher LSD post hoc, p < 0.05), parameters of the *Noctiluca* from the same food treatment show significant difference after 1 d incubation (before starvation) and after 6 h starvation are labeled in **bold**.

### Elemental composition and homeostatic regulation of *Noctiluca* in response to different quality food

Consumption of nutrient-balanced and N-limited prey for 1 day resulted in a slight loss of N content, but higher C and P contents in *Noctiluca* cells (Table 3). In contrast, ingestion on P-limited prey caused large reductions of all elements in *Noctiluca* compared to the initial starved condition, differing significantly from those fed on nutrient-balanced and N-limited prey. Generally, *Noctiluca* fed upon N- or P-limited prey significantly reduced both the amounts and fractions of the intracellular NH_4_^+^ and PO_4_^3−^ compared to those cells fed on nutrient-balanced prey and the initial starved cells (ANOVA, Fisher LSD post hoc, p < 0.05). Consumption of nutrient-balanced prey enhanced the amounts of NH_4_^+^ and PO_4_^3−^ in *Noctiluca* cells, and also the proportion of NH_4_^+^ in its total N pool (as the percentage of total cellular N). The proportion of PO_4_^3−^ (as the percentage of total cellular P) was reduced after feeding, but still comprised the major part of P in *Noctiluca*, usually >60%, except for those cells grown under P limitation where it comprised ~40% of total cellular P. The molar NH_4_^+^ to PO_4_^3−^ ratio was highest in the *Noctiluca* cells grown on P-limited prey, while it was lowest in the cells grown on N-limited prey (Table 3).

**Figure 2:**
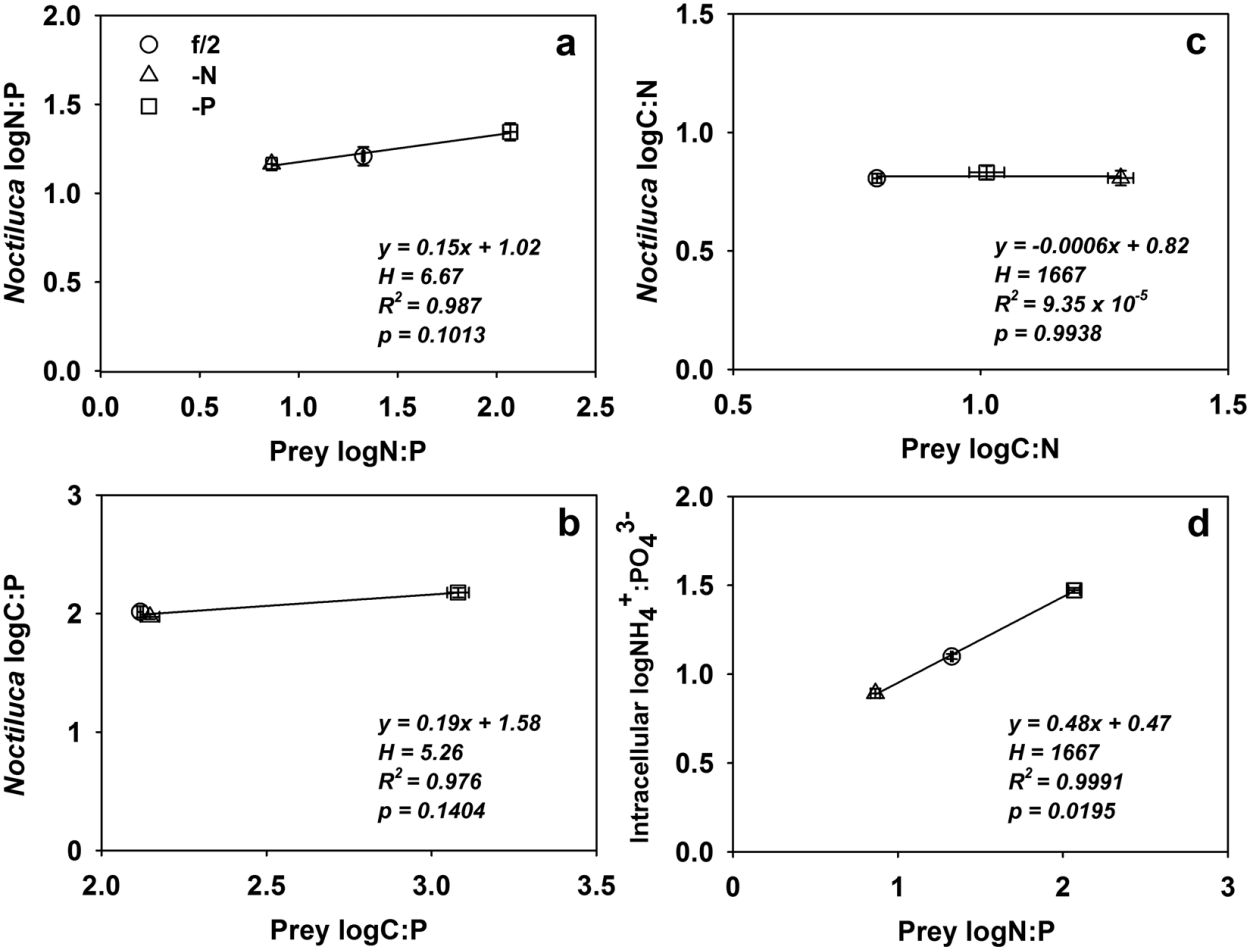
Regressions of log-normalized resource and consumer elemental ratios for *Noctiluca scintillans*: (**a**) N:P; (**b**) C:P; (**c**) C:N; (**d**) intracellular NH_4_^+^: PO_4_^3−^. 1/H is the slope of the regression between log-normalized resource and consumer elemental ratios. H = 1/slope. Error bars = ± 1 SD, n = 3.

In all cases, the elemental ratios of *Noctiluca* showed less variability than those of the algal prey upon which *Noctiluca* fed (Fig. 2). Based on the categories set by Persson *et al*.^30^ defining conditons of homeostasis^30^, N:P, C:N and C:P ratios of *Noctiluca* were strictly homeostatic (Table 3). Nevertheless, on the basis of its C:N ratio, *Noctiluca* was less variable than was the case for its N:P or C:P ratios, as it showed no significant differences after different food treatments (Table 3, Fig. 2a–c; ANOVA, Fisher LSD post hoc, p < 0.05). In addition, stoichiometric coefficients indicated a weak internal stoichiometric regulation of the NH_4_^+^ to PO_4_^3−^ ratio in *Noctiluca*, with a coefficient value of 0.48 (Table 3, Fig. 2d).

After the 1-day incubation of *Nocticula* with variable prey, experimental treatments were subjected to a subsequent 6 h starvation period. During this time, the amounts of total cellular C, N and P and the intracellular NH_4_^+^ and PO_4_^3−^ nutrients in the *Noctiluca* cells previously reared on nutrient-balanced and N-limited prey generally decreased (except the cells previously fed on N-limited prey, of which the amount of NH_4_^+^ increased after starvation), but those previously grown on P-limited prey showed no significant changes in their C and P pools (Table 3; Student’s t-test, n = 6, p < 0.05). Both the proportion and molar ratios of NH_4_^+^ and PO_4_^3−^ in *Noctiluca* cells generally increased after 6 h starvation, except for those cells that had been previously grown on P-limited prey, in which case both the total cellular elements and intracellular dissolved inorganic elements had decreased significantly after 1 day feeding (Student’s t-test, n = 6, p < 0.05).

### Excretion of *Noctiluca* from model prediction and starvation experiment

**Figure 3:**
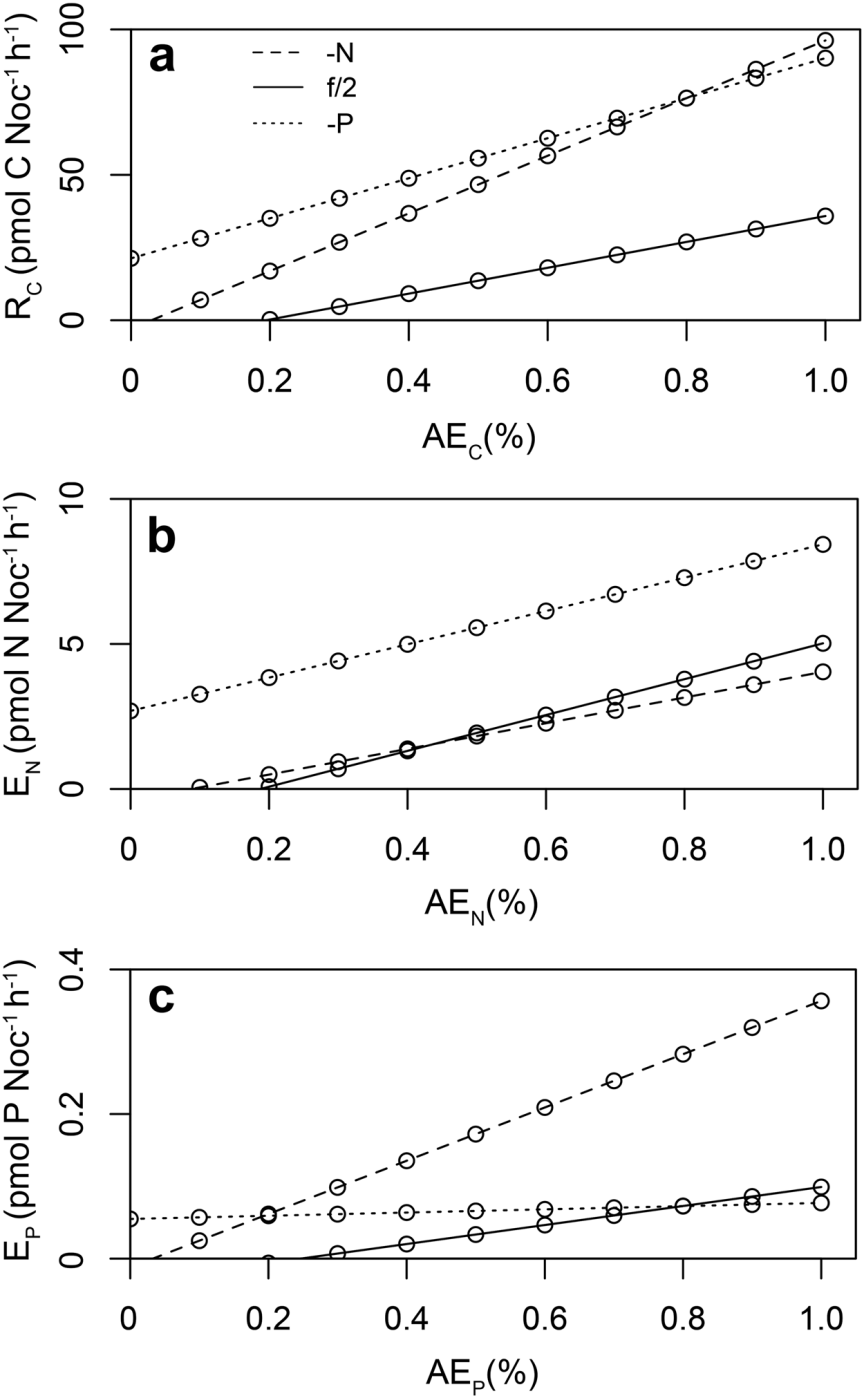
Predictions of the excretion rates of (**a**) C, (**b**) N and (**c**) P of *Noctiluca scintillans* (pmol Noc^−1^ h^−1^) grown on N-limited (-N), nutrient-balanced (f/2) and P-limited (-P) prey as a function of their assimilation efficiencies for these elements using elemental budget models.

A simple budgetary model was used herein to describe excretion rates of the biogenic elements (i.e. C, N and P) as a function of C metabolism, growth efficiency, and elemental ratios by assuming that ingested material must be assimilated by the organism before it is used for growth or metabolism^31^. Model simulations showed that excretion rates of C, N and P in *Noctiluca* were a function of their assimilation efficiencies, and were highly affected by food quality (Fig. 3). Generally, *Noctiluca* fed on nutrient-limited prey had a higher excretion rate of C and the elements that were in surplus for the growth of its prey. Note that *Noctiluca* cells grown on nutrient-balanced prey usually released less C and P than when grown on the two nutrient-limited prey conditions, and the cells grown on P-limited prey showed low but constant excretion of P regardless of the assimilation efficiencies. Assuming that the assimilation efficiency of C was 60% for *Noctiluca* in all food treatments, 80% on the unlimited nutrient, and 100% on the limited nutrient, which are the usual ranges for many feeding micro- and mesozooplankton^14, 32, 33^, *Noctiluca* grown on nutrient-balanced prey was estimated to excrete at a rate of 18.04 pmol C Noc^−1^ h^−1^, 3.79 pmol N Noc^−1^ h^−1^ and 0.07 pmol P Noc^−1^ h^−1^. Assuming these excretion rates were constant, the C, N and P contents excreted by *Noctiluca* would account for about 41%, 52% and 21% of those they ingested in 1 day. Elemental excretion rates of *Noctiluca* on nutrient-balanced prey were much lower than those nutrient-limited prey, especially P-limited prey, of which the elements released were even higher than those from ingestion (Table 4).

**Table 4:** Computational and experimental excretion rates of C, N and P (R_C_, E_N_ and E_P_, pmol Noc^−1^ h^−1^) and percentages of the excretion to ingestion (%) of *Noctiluca scintillans* grown on N-limited (-N), nutrient-balanced (f/2) and P-limited (-P) *Thalassiosira weissflogii*.

Parameters	Mode predation			Experimental excretion rates (6 h starvation)		
	-N	f/2	-P	-N	f/2	-P
R_C_ (pmol C Noc^−1^ h^−1^)	55.56	18.04	62.6	91.83 ± 8.33	92.42 ± 23.52	–
E_N_ (pmol N Noc^−1^ h^−1^)	4.04	3.79	7.28	15.65 ± 4.97	18.54 ± 5.05	−5.70 ± 3.41
E_P_ (pmol P Noc^−1^ h^−1^)	0.21	0.07	0.08	0.87 ± 0.45	0.91 ± 0.51	–
R_C_/I_C_ (%)	57.05	40.55	90.92	23 ± 3	52 ± 18	–
E_N_/I_N_ (%)	77.53	52.1	107.88	72 ± 25	64 ± 23	−21 ± 14
E_P_/I_P_ (%)	29.28	21.43	134.69	31 ± 16	67 ± 41	

Note: In the starvation experiment, percentages of the excretion to ingestion (%) of *Noctiluca* were calculated by dividing the elemental excretion in 6 h to 1-day ingestion and assuming that *Noctiluca*’s assimilation efficiency was 60% for C content in all food treatments, 80% for the excessive nutrient and 100% for the limited nutrient.

Dashes mean no significance difference of the element content of *Noctiluca* before and after starvation.

In contrast to these mass balance estimates, in the 6 h starvation experiment, *Noctiluca* previously reared on nutrient-balanced and N-limited prey generally had similar and higher excretion rates than those reared on P-limited prey, as well as those obtained from model predictions (Table 4). The amount of C, N and P content excreted by the *Noctiluca* cells previously fed on nutrient-balanced prey during 6 h starvation experiment accounted for an average of 52%, 64% and 67% of those they ingested in 1 day, much higher than those derived from modeling predictions, as well as the amount of elements in food remnants (Tables 1 and 2). Moreover, no significant excretion of the C, N and P contents was observed for the *Noctiluca* cells previously fed on P-limited prey in the starvation experiment.

## Discussion

### Stoichiometry of *Noctiluca*

By examining the balances between the elemental ratios of *Noctiluca* and those of *T. weissflogii*, *Noctiluca* appears to be strictly stoichiometrically regulated. It has a higher degree of stoichiometric regulation than some other phagotrophic flagellates, e.g. *Oxyrrhis marina* and *Gyrodinium dominans* ^6, 34, 35^. Such stoichiometric characteristic may provide *Noctiluca* cells with a competitive fitness advantage when resources are scarce or during periods of starvation^6^. Previous studies have shown that *Noctiluca* can survive without food for more than 3 weeks and that it requires a threshold of only 15 µg C L^−1^ *T. weissflogii* to maintain positive growth^36^. Nevertheless, the C:N ratio of *Noctiluca*, like other phagotrophic flagellates, is less variable than that of the N:P or C:P ratios^6, 15, 37^. In addition, *Noctiluca* appears to engage in compensatory feeding on N-limited prey (Fig. 1a,b), presumably in order to derive enough N to maintain stoichiometric balance, meeting the N requirement for basic metabolism^29^. *Noctiluca* thus displays stronger regulation of its N content than P content, i.e. relatively greater flexibility in regulating P accumulation. In other words, *Noctiluca* is more vulnerable to P limitation, which is in consistent with previous findings that dinoflagellates usually have high P demand due to their high DNA content in cell^38–40^. This result also indicates that a P limitation signal from the autotrophic phytoplankton would transfer to *Noctiluca*, but effects would be moderated.

The intracellular NH_4_^+^ and PO_4_^3−^ accounted for substantial amount of the N and P pools of *Noctiluca* (>32% and >40% respectively, Table 3), but *Noctiluca* was only able to perform a weak regulation of the molar ratio of dissolved NH_4_^+^ and PO_4_^3−^ over varying resource N:P ratios. It is still unclear what processes and mechanisms are involved in the accumulation of these dissolved inorganic contents in *Noctiluca* cells, but such processes are important in regulating cell osmosis and buoyancy^41, 42^. A weak regulation of NH_4_^+^ and PO_4_^3−^ by *Noctiluca* indicates a flexible stoichiometry with respect to its unbound nutrients, as well as osmotic and/or buoyancy regulation, and also suggests that *Noctiluca* could differentially allocate its organic and inorganic N and P pools to maintain overall homeostasis, making the concept of homeostasis regulation even more complex than previously envisioned^2^. Clearly, additional data encompassing a better understanding of NH_4_^+^ and PO_4_^3−^ accumulation processes is necessary before a more complete evaluation of the stoichiometric regulation in *Noctiluca* can be made.

Furthermore, *Noctiluca* cells grown on nutrient-balanced or N-limited prey had a significant yield in terms of C and P contents (Table 3, Fig. 1c), indicating accumulation of these two elements during feeding. This has implications for the cells also in terms of reproduction. *Noctiluca* seems to have high requirement of C and P for cell growth. In contrast, *Noctiluca* grown on P-limited prey not only resulted in considerable loss of cell elements (Table 3, Fig. 1c), but even cell death^29^. It is known that P is the structural element in the RNA and DNA skeleton, and is also required in the ATP-ADP system that are all directly related to cell division and growth^43, 44^. Therefore, inefficient housekeeping of cellular elements under stronger P deficiency might imply that P limitation disturbed the normal cell functions of *Noctiluca* metabolism and growth, making it vulnerable to direct P limitation.

### Excretion of *Noctiluca* from model prediction and starvation experiment

The measurement of elemental excretion by a feeding zooplankton in natural conditions is a technically difficult task^45, 46^. The primary difficulties come first, from the fact that co-existing phytoplankton can rapidly remove released nutrients from the dissolved pool^46, 47^. Second, *Noctiluca*, as is the case with many flagellates, is fragile and easily damaged during manipulation. The few attempts to study nutrient excretion rates of *Noctiluca* have focused on the inorganic nutrients NH_4_^+^ and PO_4_^3−^, and rates were measured without food^23, 48^. However, this sort of measurement may only determine the basal metabolic release of a starving grazer. Such measurements are also not necessarily representative of the actual excretion rate(s) from actively feeding organisms, as nutrients may also be released from the metabolic processes that are associated with feeding itself besides basal metabolism, or nutrients may be more efficiently retained in cells for actively feeding organism for reproduction rather than directly released^2, 45^. Moreover, the elements released by *Noctiluca* after metabolism are not only in oxidized (CO_2_, PO_4_^3−^) or reduced (NH_4_^+^) inorganic forms; other possible dissolved organic forms, e.g. free amino acids^49^, phosphonates and P-esters (DOP)^50^ and carbohydrates or dissolved organic C^51^, associated with food vacuole egestion and mucus formation, may also contributed substantially to C, N and P release by *Noctiluca* ^26^. As it is difficult to directly measure all these nutrient components, an elemental budget model can help to overcome these difficulties^2, 31^ and provide a general insight to the magnitude of these fluxes.

The most important implication of the elemental budget model is that the excretion rate of an element in *Noctiluca*, analogous to other zooplankton, depends largely on its physiological conditions, as well as the chemical nature of its prey^11, 31^. *Noctiluca* has nearly Redfield N:P and C:P ratios, and lower ratios than those of *T. wessfogii* under nutrient-balanced condition (Tables 1 and 3). In the framework of stoichiometry, the grazer tends to retain P and preferentially recycle N, a phenomenon that was shown in both the growth yield in the 1-day incubation and in the elemental excretion in the starvation experiment in this study. In addition, the released elements, in particular C and N, usually accounted for a significant fraction of the total metabolic budget of *Noctiluca* (41–91% and 52–107% for C and N, respectively), indicating that the cost of the growth of an actively feeding *Noctiluca*, in terms of C and N, is considerable. Obviously, even though applied here in the budget model, it is imprecise to assume an assimilation efficiency (AE) for C of 60%, and of the non-limiting nutrient of 80% and of the limiting nutrient of 100%, even though these are the typical values for many feeding micro- and mesozooplankton^14, 32, 33^. Herbivores, for example, may adjust their AE of each element, by decreasing the efficiency with which they assimilate C-rich compounds during digestion^52, 53^. Therefore, it is important to consider the different assimilation efficiencies of each element in *Noctiluca* with respect to its nutritional status in future energetic studies.

In the starvation experiment, the *Noctiluca* cells that previously fed on nutrient-balanced and N-limited prey exhibited similar excretion rates of C, N and P, rates substantially higher than the model predictions (Table 4). A mismatch of the excretion rates obtained from the model prediction and the starvation experiment suggest that growth (from model prediction) and maintenance (from starvation experiment) of *Noctiluca* may have a different set of elemental demands, with maintenance seeming to have higher requirements for all biogenic elements^2, 34^. Results of the starvation experiment also further suggest that P limitation might have disturbed *Noctiluca* basal metabolism, as there was no elemental excretion by the *Noctiluca* cells previously grown on P-limited prey.

Both the computational and experimental excretion rates of N and P reported herein are in the ranges of those reported in previous studies when NH_4_^+^ and PO_4_^3−^excretion rates were determined before and after starvation (Supplementary Table S3). For example, Drits *et al*.^48^ showed that the averaged excretion rates of *Noctiluca* in a day were 0.74 ± 0.04 pmol N cell^−1^ h^−1^ and 0.34 ± 0.02 pmol P cell^−1^ h^−1^. Ara *et al*.^23^ found that the excretion rates of *Noctiluca* decreased rapidly with time, but the highest rates found in the first 1 h were 243 pmol N cell^−1^ h^−1^ and 24 pmol P cell^−1^ h^−1^, which they thought were less influenced by starvation and closer to the actual excretion rate. Variation in the excretion rates obtained in the present and previous studies may be due to the differences in the cell size and physiological condition of the *Noctiluca* cells tested (Table 2, Supplementary Table S2). Besides, higher rates for the specimens in a short period may be also because of the increased activity during manipulation, while lower rates may result from prolonged starvation^23^. Furthermore, our study reveals that the amount of C, N or P reduced in *Noctiluca* cells after 6 h starvation was much higher than those retained in the food remnant, and the proportion of NH_4_^+^ and PO_4_^3−^ in *Noctiluca*’ N and P pools generally increased with starvation. Therefore, the elements excreted in the starvation experiment were not only ascribed to the digestion of food remains, but also to the metabolism of its cellular organic matter, and the intracellular NH_4_^+^ and PO_4_^3−^ nutrients were possibly metabolic products^54^. Measurement of elemental excretion rates by determining the nutrients excreted in the experimental bottle, and culturing the grazer in the starved condition, clearly cannot reflect the actual excretion rates of an actively feeding organism^45^.

### Roles of *Noctiluca* in nutrient recycling

Based on our model predictions, N and P excretion rates of actively feeding *Noctiluca* are lower than those reported for micro- and mesozooplankton with the same C biomass or dry weight, assuming C weight to be 43.9% of dry weight for *Noctiluca* (Table 4, Supplementary Table S2)^55, 56^. Therefore, the more significant role for *Noctiluca* as a nutrient regenerator and supplier ascribes to its extremely high concentration of NH_4_^+^ and PO_4_^3−^ in its cell, which herein accounted for 32–63% and 40–68% of its total N and P contents, depending on its physiological condition. These dissolved inorganic nutrients have been shown to contribute considerably to the N and P pools in natural assemblages^24–26, 57^. For example, Pithakpol *et al*.^58^ showed that in Seto Inland Sea, Japan, NH_4_^+^ and PO_4_^3−^ contained in *Noctiluca* cells contributed up to 119% of the N pool and 80% of the P in the water column (0–35 m depth). This is especially true when *Noctiluca* blooms are formed, as the cells at this stage stop feeding as they go into stationary growth, becoming starved and/or nutrient limited, and their mortality rate increases. Liberation of NH_4_^+^ and PO_4_^3−^ and other nutrients of these cells would stimulate the growth of phytoplankton species living near the red tide patches and improve the food quality for *Noctiluca* again^19, 20, 24, 26^. Besides the inorganic nutrients NH_4_^+^ and PO_4_^3−^, *Noctiluca* also contains high amounts of organic substances in cell^26, 27^. Decaying *Noctiluca* cells, therefore, would contribute organic matter for bacteriovorous protozooplankters and could supply food to actively feeding *Noctiluca* ^26, 27^. In addition, *Noctiluca* excretes mucus to trap food items, and the decomposition of this organic matter by heterotrophic marine bacteria would fuel the microbial loop and result in an increase of recycled nutrients^26, 59^. Therefore, the lysis of *Noctiluca* cells and subsequent mineralization activity could be a significant source of inorganic and organic nutrients fueling further phytoplankton production, prolonging the bloom duration and existence of *Noctiluca* ^19, 20, 26, 58^. For example, Schaumann *et al*.^26^ found that the marked release of nutrients, especially NH_4_^+^ and PO_4_^3−^ by *Noctiluca* contributed to autochthonous eutrophication in the German Bight, initiated by diatoms bloom, e.g. *Rhizosolenia sbrubsolei*, *R. setigera* and *Guinardia flaccida* that are then fed upon by *Noctiluca*. Harrison *et al*.^19^ conceptualized *Noctiluca* as an offshore manifestation of eutrophication since it feeds on the phytoplankton bloom caused by anthropogenic nutrients.

The interaction between *Noctiluca* and phytoplankton is not a simple mutually supportive relationship^20, 23, 26, 58^. Rather, *Noctiluca* accumulates and releases each element with different efficiencies according to its physiological status (the present study), which would intensify the effect of nutritional imbalances in primary producers and then strengthen the trophic feedback to *Noctiluca*. As stated above, *Noctiluca* weakly regulates its intracellular NH_4_^+^ to PO_4_^3−^ ratio, and it is an efficient recycler of N, but recycles P with a low efficiency, thus it would usually suffer P limitation. This would intensify P-limitation from phytoplankton to *Noctiluca*, causing a negative impact on it. Our findings may provide an important clue for the population dynamics of *Noctiluca* in the field. For instance, the occurrence of *Noctiluca* in Hong Kong waters has a strong seasonality, with high abundance in winter–spring, while almost no occurrence in summer–fall^60^. The Hong Kong coastal waters, especially the eastern waters, could potentially experience N-limitation in winter and spring^61^. A possible explanation for its high abundance and long residence, besides the suitable temperature during winter-spring, is that *Noctiluca* is less susceptible to N-limitation and able to efficiently recycle N. In contrast, in summer and fall, the diatom-dominated phytoplankton assemblages in Hong Kong waters become proportionately more P limited^60, 62, 63^, and the reduction of the P content in phytoplankton could be intensified through *Noctiluca*’ feeding activity, which would be detrimental to *Noctiluca*, leading to its disappearance in the water column^29^.

In conclusion, *Noctiluca* is nearly homeostastic, but it regulates its internal N more strongly and efficiently than its P content, thus is more vulnerable to P-limitation. It is able to differentially allocate N and P to organic and inorganic pools to maintain overall homeostasis. Excretion of C, N, and P by *Noctiluca* is coupled through processes that help to maintain its elemental composition, and also depends highly on resource nutritional quality. *Noctiluca* seems to have a shifting role in planktonic food web, from mainly exerting top-down control during most of its pelagic life to fuelling bottom-up processes due to the liberation of intracellular nutrients during senescence.

## Materials and Methods

### Preparation of experimental organisms

An initial inoculum of cells of *Noctiluca* was gently collected from Port Shelter in eastern Hong Kong in October 2011 using a plankton net of 120 µm mesh size. Cells were isolated and maintained in culture as described by Zhang *et al*.^29^ in a temperature-controlled chamber at 23 ± 1 °C on a 14:10 h L/D cycle with 20 µmol m^−2^ s^−1^ illumination.

The diatom *T. weissflogii* was used as prey. Nutrient-balanced *T. weissflogii* was obtained using a batch culture with f/2 + Si medium^64^. N-limited and P-limited *T. weissflogii* were achieved by growing cells in f/2 + Si medium with a 40-fold reduction in N (final conc. 22.05 μM NO_3_^−^N) or P (final conc. 9.05 μM PO_4_^3−^P), respectively. The seawater used for preparing the media was the surface water collected from Port Shelter, and aged before usage (the concentrations of N and P in the seawater were negligible compared to the limiting nutrients in the media). Algal cultures were maintained at the same conditions as described above with illumination of 80 μmol photons m^−2^ s^−1^. Experiments were started once the cultures reached stationary phase. All cultures were sampled for cell counts and for cellular C, N and P analyses before the experiments were started.

### Experimental manipulations

Experiments were designed to determine the stoichiometric regulation and element budgets of *Noctiluca* when facing different quality food. To avoid any potential effects of food carryover, *Noctiluca* used in this study were gently washed and resuspended with 0.2 µm-filtered autoclaved seawater 24 h prior to the experiment to void the food vacuoles. Feeding experiments involved 3 different *T. weissflogii* cultures (cultures grown in N-limited, nutrient-balanced and P-limited media) that were diluted to 1.5 × 10^4^ cells mL^−1^ with appropriate amount of autoclaved filtered seawater. *Noctiluca* cells were inoculated into these food suspensions (2 L) in triplicate with a final concentration of ~13 *Noctiluca* cells mL^−1^. Duplicate bottles with each type of prey were used as the controls. All bottles were incubated at dim light (~10 μmol photons m^−2^ s^−1^) under the same conditions described above. To avoid cell aggregation or settlement, cultures were gently agitated manually 2–3 times a day. Preliminary trials using a plankton wheel (at various rotation speeds) significantly reduced the rate of growth of *Noctiluca* (data not shown), and thus the best growth of predator and prey was achieved through regular manual agitation.

After 1 d incubation, duplicate 20 mL aliquots were withdrawn from the bottles with 25 mL plastic long pipettes for determining *Noctiluca* abundance, and 1 mL aliquots were collected with pipette (Eppendorf) for ultimately determining prey abundance. Both aliquots were preserved with acid Lugol’s solution (final conc. 2%). The preserved cells of *Noctiluca* were also measured to determine cell size (first 100 cells encountered) using the SPOT image program (Version 3.5.0). All measured cells were examined for the presence of prey.

The remaining *Noctiluca* cells were collected on a mesh with a size of 100 μm, and gently washed and resuspended with autoclaved seawater to initiate excretion experiments. The resuspended *Noctiluca* cells of each food treatment (N-limited, nutrient balanced and P-limited) were immediately transferred to 3 (triplicate), 150 mL polycarbonate bottles, each yielding a concentration ~ 50 cells mL^−1^, and the bottles were filled with autoclaved filtered seawater and incubated under the same condition as in the feeding experiment for 6 h. Subsamples for total cellular C, N and P contents, as well as the intracellular dissolved nutrient contents NH_4_^+^ and PO_4_^3−^ in *Noctiluca* cells were collected and determined as stated below before and after 6 h incubation. The 6 h incubation period was based on previous data showing that *Noctiluca* can void its food vacuoles in 4.5 to 7 h after feeding for 1 d^65–67^.

### Analytical measurements

Before the grazing experiment started, samples for analyses of cellular C, N, and P of differently grown *T. weissflogii* were taken from the respective culture bottles by filtering 15 to 25 mL cultures onto pre-combusted (550 °C, 4 to 5 h) GF/C glass-fiber filters. Samples for determining total elemental composition (C, N and P) and intracellular nutrients (NH_4_^+^ and PO_4_^3−^) of *Noctiluca* were collected at the beginning and end of the feeding experiment, and also after 6 h starvation. Usually more than 1200 *Noctiluca* cells were filtered on pre-combusted 25 µm GF/C filters for cellular C, N and P analyses. A similar amount of *Noctiluca* cells were filtered onto a 20-μm PC membrane and the filters were immediately submerged into 10 mL MilliQ, sonicated (50/60 hz, 20 min), and then filtered through a 0.2 µm disk filter. The filtrate was used to determine the amount of dissolved inorganic NH_4_^+^ and PO_4_^3−^ in *N. scintllans*. Subsamples for measuring these elements were in triplicate.

Cellular C and N were analyzed with a CHN elemental analyzer (Perkin-Elmer) and cellular P was analyzed as orthophosphate after acidic oxidative hydrolysis with 1% HCl^68^. Analyses of NH_4_^+^ and PO_4_^3−^ were conducted manually according to Strickland and Parsons (1972)^69^, and the detection limit was 0.5 µmol L^−1^ for PO_4_^3−^ and 0.5 µmol L^−1^ for NH_4_^+^.

### Rate process and elemental composition calculations

Clearance (*F*, µL Noc^−1^ d^−1^) and ingestion (*I*, cells Noc^−1^ d^−1^) rates were calculated according to Harris *et al*.^70^ and Frost^71^, respectively:

1 $$F=\,{\rm{ln}}({C}_{t}\text{'}/{C}_{t})\times (V/nt)$$

2 $$I\,=\,F\times [C]$$

where *C*′_*t*_ and *C*_*t*_ (cells mL^−1^) are the prey concentrations at the end of the incubation in control and experimental bottles, respectively; *V* is the volume of the culture (mL), *t* (d) is the incubation period and n is the number of *Noctiluca* used; [*C*] is the prey concentration in the experimental bottle averaged over the incubation period.

The homeostasis coefficient, *Η*, was calculated as:

3 $$H\,=\,\frac{{\mathrm{log}}_{10}(x)}{{\mathrm{log}}_{10}(y)-{\mathrm{log}}_{{\rm{10}}}(c)}$$

where x is the resource nutrient stoichiometry, *y* is the organism’s nutrient stoichiometry and c is a constant^2^. Therefore, 1/H is the slope of the regression between log(x) and log(y) which is based on values between zero and one. According to Persson *et al*.^30^, if the regression relationship is non-significant (p > 0.1), the organism is considered ‘strictly homeostatic’ and an organism with 1/H = 1 is not considered to be homeostatic. Homeostatic plots with significant regressions and 0 < 1/H < 1 are classified as: 0 < 1/H < 0.25 ‘homeostatic’, 0.25 < 1/H < 0.5 ‘weakly homeostatic’, 0.5 < 1/H < 0.75 ‘weakly plastic’, 1/H > 0.75 ‘plastic’. In the present study, estimations of the homeostatic coefficient *H* for *Noctiluca* were only considered for *Noctiluca* cells collected immediately after 1 d incubation.

Determination of the stoichiometric composition and elemental growth yield of *Noctiluca* in term of C, N and P were corrected by subtracting the amount of elements of the food remnant in *Noctiluca* (assuming those prey items were intact and undigested, and an assimilation efficiency for C of 60%, and that of the non-limiting nutrient was 80% and that of the limiting nutrient was 100% for the remaining prey items). Elemental growth yield was calculated by comparing the elements per individual cell before and after 1 d incubation, and gross growth efficiency in terms of C (GGE_C_) was calculated by dividing the growth yield by the ingestion per *Noctiluca* in terms of C. Based on the assumption that ingested material must be assimilated by organism before it is used for growth or metabolism, the expression for N and P excretion rates as a function of C metabolism, C-based growth yield, and C to nutrients ratios can be derived from elemental budget models modified from Landry^31^:

4 $${\rm{RC}}={{\rm{AE}}}_{{\rm{C}}}\times {{\rm{I}}}_{{\rm{C}}}-{{\rm{G}}}_{{\rm{C}}}$$

5 $${E}_{{\rm{N}}}=\frac{{{\rm{AE}}}_{{\rm{N}}}\times {{\rm{I}}}_{{\rm{C}}}}{{{\rm{C}}:{\rm{N}}}_{{\rm{prey}}}}-\frac{{{\rm{G}}}_{{\rm{C}}}}{{{\rm{C}}:{\rm{N}}}_{{\rm{pred}}}}$$

6 $${{\rm{E}}}_{{\rm{P}}}=\,\frac{{{\rm{AE}}}_{{\rm{P}}}\times {{\rm{I}}}_{{\rm{C}}}}{{{\rm{C}}:{\rm{P}}}_{{\rm{prey}}}}-\frac{{{\rm{G}}}_{{\rm{C}}}}{{{\rm{C}}:{\rm{P}}}_{{\rm{pred}}}}$$

where AE_C_, AE_N_ and AE_P_ is the assimilation efficiency of C, N and P (as a percentage); I_C_ (pmol C Noc^−1^ h^−1^) is the ingestion of C; R_C_ (pmol C Noc^−1^ h^−1^) indicates a combination of C respiration and other forms of C excretion, E_N_ (pmol N Noc^−1^ h^−1^) and E_P_ (pmol P Noc^−1^ h^−1^) are the excretion of N and P related nutrients; G_C_ is C-based growth yield (pmol C Noc^−1^ h^−1^); C:N_prey_ and C:P_prey_ are C:N and C:P of prey; C:N_pred_ and C:P_pred_ are C:N and C:P of predator.

Rates of nutrients excretion were estimated by comparing the elemental contents per individual cell before (right after 1 d feeding experiment) and after 6 h starvation, and only those that had significant differences were reported.

### Statistical analysis

Distributions of the data (log transformed before analysis as necessary) were evaluated by Shapiro-Wilk test before analysis of variance (ANOVA) and post hoc comparisons. Data that were normally distributed were analyzed using standard one-way ANOVA and Fisher LSD’s post hoc comparisons with significance levels of p < 0.05. Comparisons between two groups were conducted using Student t-test (2-tailed) with significance levels of p < 0.05. All analyses were conducted using Sigma Plot 11.0 (Systat Software Inc., San Jose, CA). The models were simulated using R software v. 3.0.2 (R Development Core Team 2013).

## Electronic supplementary material

Supplementary information
