# Supplementary material for: Effects of prey of different nutrient quality on elemental nutrient budgets in Noctiluca scintillans
Source: Sci Rep. 2017 Aug 8;7:7622. doi: 10.1038/s41598-017-05991-w (PMC5548927; doi:10.1038/s41598-017-05991-w)
Supplement: Supplementary file 1 — Supplementary information [file 41598_2017_5991_MOESM1_ESM.doc]

*The following supplement accompanies the article*

**Effects of prey of different nutrient quality on elemental nutrient budgets in *Noctiluca scintillans***

**Shuwen Zhang^1^, Hongbin Liu^1, *^, Patricia M. Glibert^2^, Cui Guo^1^, Ying Ke^1^**

*^1^Division of Life Science, Hong Kong University of Science and Technology, Clear Water Bay, Hong Kong SAR*

*^2^University of Maryland Center for Environmental Science, Horn Point Laboratory, P.O. Box 775, Cambridge, MD 21613, USA*

*Corresponding author: fax +852 2358 1559; email: [liub@ust.hk](mailto:liub@ust.hk)

**Running title: Feeding and nutrient regulation in *Noctiluca***

Table S1 The abundance of *N. scintillans* before and after 1-day incubation (×10^3^ cells L^-1^)

| Treatment | Initial | After incubation |
| --- | --- | --- |
| f/2 | 13.37±1.02 | 14.03±0.72 |
| -N | 14.25±1.78 | 15.58±1.48 |
| -P | 13.45±0.51 | 12.75±0.91 |

Table S2 The amounts of intracellular NH_4_^+^ and PO_4_^3-^  and the excretion rates of *Noctiluca scintillans* determined in previous studies.

| Cell size  (µm) | Intracellular N  (pmol cell^-1^) | Intracellular P  (pmol cell^-1^) | N excretion rate (pmol cell^-1^ h^-1^) | P excretion rate  (pmol cell^-1^ h^-1^) | Respiration (pmol O2 cell^-1^ h^-1^) | Method | Reference |
| --- | --- | --- | --- | --- | --- | --- | --- |
| 448–1019.2 | 1,000–11,200 | 10–150 |  |  |  | Based on calculation | Pithakpol et al. 2000 |
| 486–749 | 1,672–6,755 | 109–618 |  |  |  | Direct measurement of the extracted nutrients | Montani et al. 1998 |
|  |  |  | 0.74±0.04 | 0.34±0.02 | 4.62±0.28 | Starvation experiment conducted in the dark at 12°C for 24h | Drits et al. 2013 |
| 325–826 | 450±10–4950±39 0 (2160±150) | 70±<10–400±10  (190 ± 10) | 2.4±0.9 to 242.6±7.4 | 0.2±0.1 t o24.2 ±10.8 |  | Direct measurement of the extracted intracellular nutrients.  Acclimated the cells to the in situ surface temperature in the incubator for 4–6 h, then measured the excretion by starvation for 1–72h | Ara et al. 2013 |
| 560 |  |  |  |  | 17,400±5,400 | Starvation experiment conducted in the dark at 26°C for 24h | Saito et al. 2006 |

**Table S3 Excretion rates (pmol Noc^-1^ h^-1^) of micro- and mesozooplankton with the same carbon biomass or dry weight with *Noctiluca scintillans* (Dolan 1997; Ikeda 1985), assuming carbon weight to be 43.9%.**

| Type | Protist | | | Mesozooplankton | | |
| --- | --- | --- | --- | --- | --- | --- |
|  | R_C_ | E_N_ | E_p_ | R_C_ | E_N_ | E_p_ |
| f/2 | 483 | 7.55 | 1.09 | 55.39 | 8.71 | 0.38 |
| -N | 469 | 7.29 | 1.05 | 52.90 | 8.36 | 0.37 |
| -P | 488 | 6.29 | 0.92 | 43.41 | 6.98 | 0.30 |
